# Environmental Determinants of the Distribution and Abundance of the Ants, *Lasiophanes picinus* and *L. valdiviensis*, in Argentina

**DOI:** 10.1673/031.008.3601

**Published:** 2008-05-09

**Authors:** Paula Fergnani, Paula Sackmann, Fabiana Cuezzo

**Affiliations:** ^1^ Laboratorio Ecotono, Centro Regional Universitario Bariloche, INIBIOMA UNC-CONICET. Pasaje Gutiérrez 1125 (8400) Bariloche, Río Negro, Argentina; ^2^ CONICET Instituto Superior de Entomología (INSUE), Facultad de Cs. Naturales e IML. Miguel Lillo 205. (4000) San Miguel de Tucumán, Argentina

**Keywords:** energy theory, productivity hypothesis, thermal limitation hypothesis, environmental gradient, Patagonia

## Abstract

The distribution and abundance variation of the terrestrial ants, *Lasiophanes picinus* and *Lasiophanes valdiviensis* Emery (Formicinae: Lasiini), which are endemic in Patagonia (Argentina and Chile), are described and a set of environmental factors are examined to explain the observed patterns. Ants were collected using 450 pitfall traps arranged in 50, 100 m^2^ grid plots each with nine traps within a roughly 150 × 150 km area representative of the subantartic-patagonian transition of Argentina. Five sampling periods each 8-days long were carried out between November 2004 and March 2006. To understand the distributional patterns and their link to environmental variables discriminant analysis was used. Path analysis was performed to test for direct and indirect effects of a set of environmental variables on species abundance variation. *L. picinus* was more frequently captured and attained higher abundance in the forests, while *L. valdiviensis* was more frequently captured and more abundant in the scrubs. The maximum daily temperature and mean annual precipitation explained *L. picinus* distribution (i.e. presence or absence) with an accuracy of 90%. *L. valdiviensis* distribution was predicted with almost 70% accuracy, taking into account herbal richness. The maximum daily temperature was the only climatic variable that affected ant abundance directly; an increase in temperature led to an increase of *L. picinus* abundance and a decrease of *L. valdiviensis* abundance. The amount of resources, as indicated by the percent plant cover, explained the variation of the abundance of both species better than the variety of resources as indicated by plant richness (i.e. models including plant richness had low fit or no fit at all). A direct effect of habitat use by cattle was found, as indicated by the amount of feces in the plots, only when variables related to the amount of resources were replaced by variables with less explanatory power related to the variety of resources. This study provides new data on the ecology *of Lasiophanes* species in relation to existing hypotheses proposed to explain patterns of abundance variation. Evidence is provided that changes in temperature (i.e. global climate change) may have important consequences on populations of these species.

## Introduction

Ants are abundant insects and are considered important in ecosystem functioning. They have diverse ecological roles, including nutrient cycling, seed dispersal and population regulation of other insects (Holldobler and Wilson 1990; Folgarait 1998). Also, several studies have shown that ant assemblages are sensitive to changes in environmental conditions (e.g. Kaspari et al. 2000a; Hoffmann and Andersen 2003; Corley et al. 2006; Sackmann and Farji-Brener 2006). However, efforts devoted to the study of ant ecology in the New World have been biased towards areas of high species diversity (e.g. the Neotropical Region, sensu Morrone 2001). Other areas, such as the Andean Region, which have much less ant diversity, have been long set apart (e.g. Bolivia and Perú, Lattke 2003). This has been also the case of the Andean Patagonia, a region that, in spite of its low species richness (around 30 species, Fergnani 2006), has a high level of endemisms (e.g. genus *Lasiophanes*, and around 70% of recorded species (Kusnezov 1959)). Also, many species within the ant fauna of NW Patagonia are basal within its subfamilies, and are less evolved than representatives of the same taxa in other regions; moreover, the whole fauna can be considered as a relict of an ancient fauna with a broader distribution (Kusnezov 1959). It has been proposed that more taxa (e.g. species with contrasting geographical ranges) should be included in ecological analyses to minimize the risk of making general statements, based on studies of only a limited number of taxa (Prodon et al. 1987). In particular, impoverished faunas such as the Patagonian assemblages represent an opportunity for systematic, biogeographic and, ecological research (Snelling and Hunt 1975).

*Lasiophanes* Emery (Formicinae: Lasiini) is a small genus of five described species that are endemic of Patagonia (Argentina and Chile) that is related to *Lasius* of the Northern Hemisphere and *Melophorus* of Australia and New Zealand (Kusnezov 1952). The taxonomy of *Lasiophanes* is poorly understood, and the group is in need of revision (Snelling and Hunt 1975). Three species have been reported for Argentina: *Lasiophanes picinus* (Roger, 1863), *L. valdiviensis* (Forel, 1904), and *L. atriventris* (Smith F, 1858) (Kusnezov 1952; Cuezzo 1998). Kusnezov (1952; 1959) only recognized two species for Argentina: *L. picinus* with two subspecies that are synonymous at present, and *L. nigriventis* which is known today as *L. atriventris*. However, Snelling and Hunt (1975) said that in their opinion material determined and recorded as *L. picinus bruchi* by Kusnezov is actually *L. valdiviensis*. Although *L. valdiviensis sensu stricto* has been recorded for Argentina only ten years ago (Cuezzo 1998), in the present work, comments made by Kusnezov (1952; 1959) on *L. picinus bruchi* will be considered as to be made on *L. valdiviensis*.

Little is known about these species. The work by Kusnezov (1952; 1959) gives some details about their distribution, habitat preferences and nesting habits. No systematic studies have been carried out after that date. It is worthy to note that *L. atriventris* has arboreal habits, living and nesting on trees. On the other hand, *L. picinus* and *L. valdiviensis* are terrestrial species (Kusnezov 1952) and have almost the same geographical distribution. The species have been found at Neuquén, Río Negro, Chubut and Santa Cruz provinces but only *L. picinus* reaches the southern tip of Patagonia at Tierra del Fuego (Cuezzo 1998). Despite the similarity in their geographical ranges, patterns of habitat use are thought to be different (Kusnezov 1978). *L. picinus* lives in humid areas of western Patagonia but it can penetrate more arid areas following the vegetation that grows along the rivers or where subterranean water makes the soil more humid (Kusnezov 1959). *L. valdiviensis* in turn, lives in more arid areas in the eastern part of the genus distribution (Kusnezov 1952). However, within the arid areas *L. valdiviensis* is thought to prefer sites with relatively humid soils (Kusnezov 1952).

The purpose of the present work was first, to describe the distribution and abundance variation of the species *L. picinus* and *L. valdiviensis. L. atriventris* was not included in this study because it has a more restricted distribution and lower abundance as shown by pitfall catches. Secondly, the ability of a set of environmental factors related to an existing ecological framework (see below) was analyzed to explain the distribution and abundance variation of the species.

Much work has been done to understand patterns of abundance variation which lead to the proposition of different hypotheses (Kaspari et al. 2000a), for example, the thermal limitation hypothesis (Kaspari et al. 2000b; Hawkins et al. 2003), and the productivity hypothesis (Currie et al. 2004). For ants in particular, Kaspari et al. (2000a) found that abundance (nests/m^2^) at a geographical scale increased primarily with net primary productivity and secondly with temperature, and seasonality. At a local scale the abundance of granivorous ants increased with summer precipitation in the Chihuahuan desert (Kaspari and Valone 2002), but temperature explained significant proportions of the variation in ant species density and abundance along an elevational gradient (Botes et al. 2006). In NW Patagonia previous studies showed that the effect of fire on ant abundance depends on the habitat structural complexity (e.g. abundance increase in complex habitats but remain unchanged in simple habitats after fire (Farji-Brener et al. 2002; Sackmann and Farji-Brener 2006). Also, disturbance by grazing and trampling, which alters the habitat, can have great influence on ant fauna in the Western Australian woodlands (Abensperg-Traun et al. 1996). At least in the open habitats of Australia, the abundance of some ant species can increase with grazing, while the abundance of others can decrease (Read and Andersen 2000).

It is difficult to predict the effects of the environmental factors on the distribution and abundance of terrestrial Patagonian *Lasiophanes* except that the hypotheses related to energy predict a positive relationship between ant abundance and the environmental predictors. On the one hand the hypotheses were formulated for understanding the abundance patterns of all species that inhabit a region and we are studying just two species. The variation in abundance of these species could not be in accordance with the predictions despite what ever the variation in abundance of the entire assemblage may be. On the other hand, although little is known about these species, we expect that the key factors that regulate their populations will be different, or if the factors are the same that they will have contrasting effects because the species are thought to live in different habitats (Sackmann and Farji-Brener 2006).

## Methods

### Study area and sampling plots

**Figure 1.**
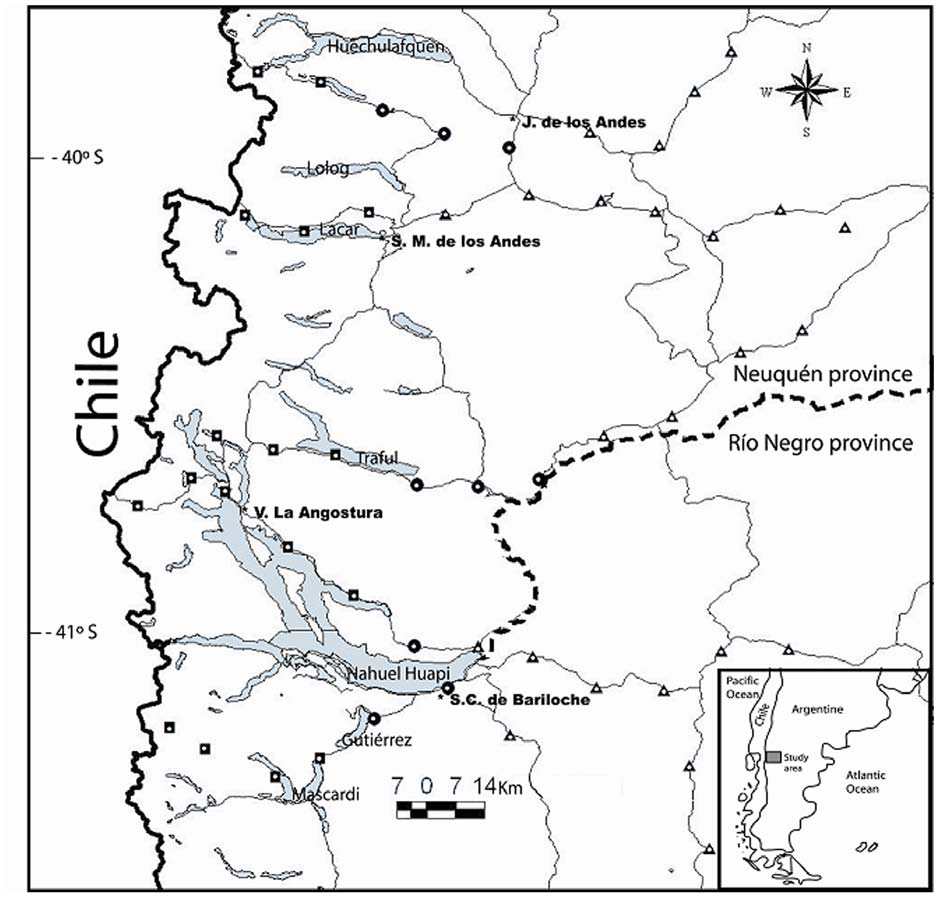
Map of the studied area indicating the localization of sampling plots, within the A) forests (squares), B) scrubland (circles) and C) steppes (triangles).

**Figure 2.**
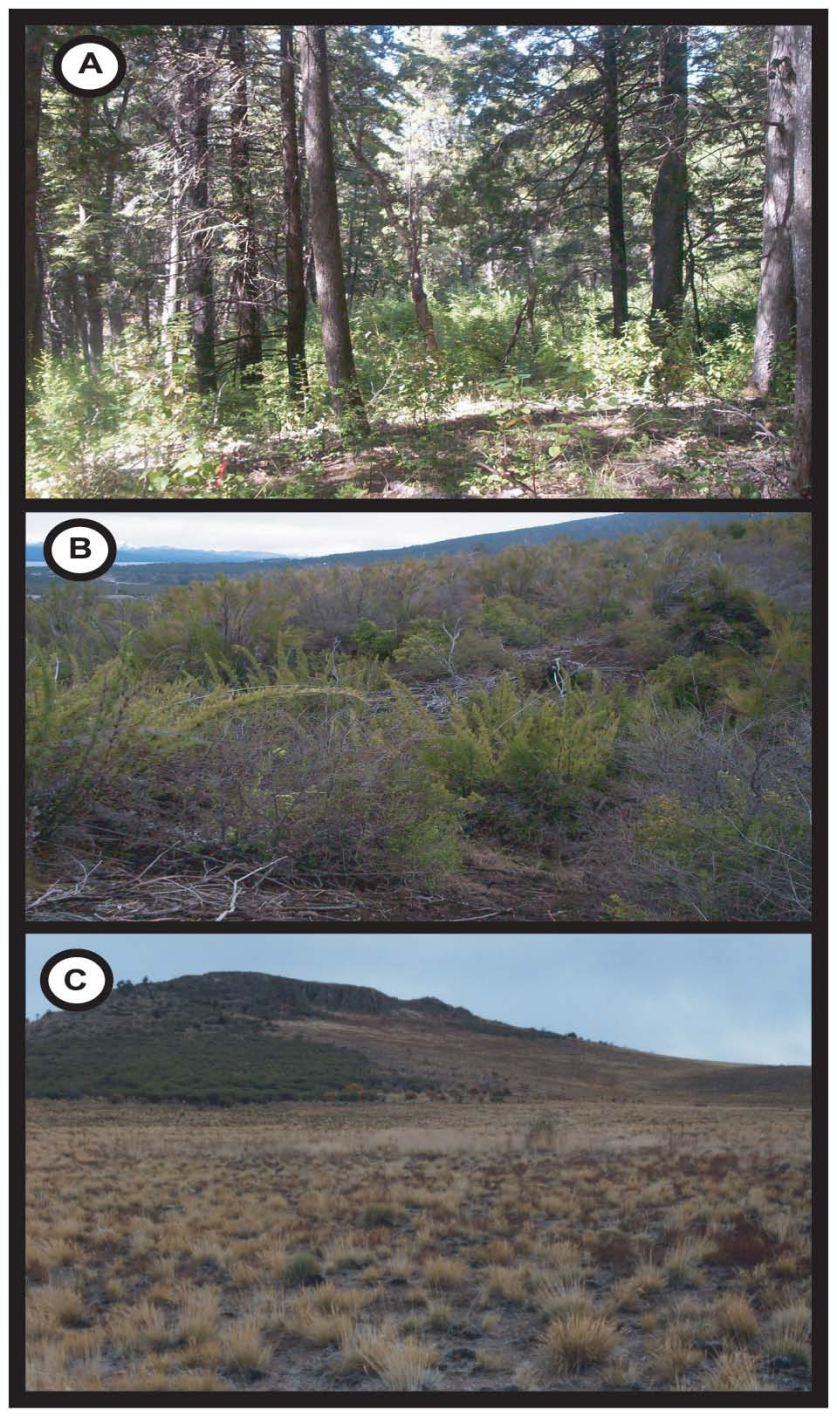
Aspect of the three sampled habitats: A) Forest, B) Scrub, C) Steppe.

The study was conducted in NW Patagonia in the Neuquén and Río Negro provinces of Argentina, between -39.8 and -41.4 S latitude and -70.3 and -71.8 W longitude in an area of approximately 28,000 km^2^ (Figure 1). Mean annual temperature is 8° C, although temperature can fluctuate from a mean minimum of -2°C in the coldest month, July, to a mean maximum of 23°C in the warmest month, January. The Andean mountains act as a barrier to the westerly airflow at temperate latitudes, resulting in a pronounced eastward rain shadow. The humid winds from the Pacific rise up and across the Andean crests causing the most intense rainfalls on the western (Chilean) side of the Cordillera and a marked W-E gradient on the eastern (Argentinean) side. The mean annual precipitation along this gradient drastically declines from > 3000 mm in the West to < 500 mm only 100 km towards the east (Barros et al. 1983). Major climatic, soil and biotic differences along the west-to-east gradient allow the distinction of three major habitat types, namely: (1) evergreen forest, in sites of 2000–1600 mm of mean annual precipitation, (2) scrub, in sites of 2000–1200 mm of mean annual precipitation, and (3) xeric steppe, in sites of 800–400 mm of mean annual rainfall (Figure 2). (Dimitri 1962). In the western zone there are temperate rainforests that are dominated by *Nothofagus dombeyi* up to 900 m above sea level. Above this altitude, the dominant species is *N. pumilio*. Semi-arid scrub vegetation grows along the foothill zone; here *N. antarctica* in its shrub life form is dominant and it's usually mixed with other shrubs such as *Diostea juncea* and *Chusquea culleou*. Small groups or single isolated trees (e.g. *Austrocedrus chilensis* and *Maytenus boaria*) also grow in this habitat. In the eastern zone, the steppe lacks tall vegetation except for small groups or isolated shrubs. It is mainly composed of short xerophytic shrubs and herbs. Dominant species include *Senecio bracteolatus, Mulinum spinosum* and *Stipa speciosa*.

Within this area 5 longitudinal roads were followed and 9 to 12 sites were selected among them for a total of 50 sampling sites, that covered the three main ecological units; forest, scrub and steppe (Figure 1). We excluded all peculiar sites (e.g. wetland meadows) in order to have a representative coverage of the region. Along each transect, sampling sites were separated by 15 km on average and all of them were GPS-located. Sites altitude was in average 880 ± 16 (SE) (range: 600 – 1000 meters above sea level). At each site one 10 × 10 m plot was established that was, in turn, subdivided into four quadrants.

### Ant abundance estimation

At each plot, nine pitfall traps were placed in a 10 × 10 m area, with 5 m spacing between traps. Traps were opened for 8 days in November 2004, January and March 2005 and January and March 2006 (i.e. 40 days total and 450 traps per day). Traps were plastic cups of 9 cm diameter, 12 cm depth partly filled with water, propylene glycol and detergent. To minimize ground disturbance while inserting and removing the contents of traps, in subsequent sampling periods, two traps were nested one inside the other at each site, and sunk into the ground with their rims touching, with the mouth of the trap level with the soil surface. In this way, only the inner container was removed to collect the contents of the cups at each sampling period. The contents from the nine traps at each plot were pooled into one sample and taken to the laboratory in ethylic alcohol 80%. Ant specimens were separated and all *Lasiophanes* species were identified and counted. We used different sources to identify species: keys by Kusnezov (1978) from original descriptions, comments made by Snelling and Hunt (1975) and comparisons with material deposited in the Kusnezov collection (Instituto-Fundación Miguel Lillo, Tucumán, Argentina).

For each sampling event we calculated the average abundance per trap per species, in order to avoid problems related with unequal sampling effort (e.g. missing traps). Then the five estimates of abundance per sampling were averaged because for one site the January 2006 sampling could not be carried out. However, the corrected abundance was highly correlated to raw abundance for both species (*L. picinus*: Pearson's r = 0.98 (p<0.001) and *L. valdiviensis* Pearson's r = 0.99 (p<0.001).

### Environmental variables included in the models

Environmental variables were chosen taking into account existing hypotheses proposed to explain abundance-energy relationships. The *productivity hypothesis* states that climate, mainly through the combined effects of rainfall and temperature, strongly affects net primary productivity which in turn limits the abundance of individuals (Kaspari et al. 2000a; Hawkins et al. 2003; Currie et al. 2004). However, abiotic factors, such as temperature, could limit access to a site's primary productivity. The *thermal limitation hypothesis* (Kaspari et al. 2000b) proposes that the direct effect of temperature on insect development, growth and behavior may contribute to regulating the abundance of individuals in an assemblage. Also, habitat heterogeneity (for example, more microhabitats available, or high plant diversity), and environmental disturbances, may shape the variation in abundance of insects across the space (Brown and Lomolino 1998; Davidowitz and Rosenzweig 1998; Siemann et al. 1998; Hoffmann and Andersen 2003). Some studies have found that increasing plant diversity increases productivity and thus abundance of consumer species that in turn may increase arthropod herbivore diversity (Niemelä et al. 1996; Siemann 1998; Siemann et al. 1998). Also, arthropod abundance may be affected (i.e. decreased and increased) when environmental disturbances change the habitats structure (Sackmann and Farji-Brener 2006).

### Estimation of environmental variables a) Vegetation cover

Vegetation cover gives a good measure of plant biomass, as an indication of the capacity of vegetation to accumulate organic material that can be used as food and/or shelter for animals (Mueller-Dombois and Ellenberg 1974.). Given that plant biomass correlates positively and strongly with net primary productivity (Evans et al. 2005 and other references therein) vegetation cover is here considered to be suitable as a productivity energy metrics throughout.

Canopy cover (all woody vascular plants > 2 m in height and >10 cm in diameter breast height), shrub cover (all woody vascular plants between 0.30–1 m in height), and herbaceous cover (all vascular plants < 0.30 m in height) were estimated. A concave spherical densiometer was used to estimate the proportion of *canopy cover* in the 17 forest and 9 scrubland plots (i.e., the 24 steppe plots lack of trees). Each 10 × 10 m plot was visually divided into four quadrants to make four densiometer readings, one in each quadrant, facing each of the four cardinal directions. The four cover values were averaged to have an overall estimation of *canopy cover* for each plot. *Shrub cover* was estimated visually for each species in the entire plot (%) and then summed up. The *herbaceous cover* was estimated for each plot by randomly throwing a 0.50 × 0.50 m wood frame subdivided into a 25- celled nylon string-grid four times. In each placement, the proportion of cells covered by herbs was estimated and then the four values were averaged to produce an overall estimation of herbaceous cover for each plot.

### b) Plant richness

It has been suggested that plant diversity should be important in determining animal diversity (e.g. Hutchinson 1959). At the community level an increment in plant richness can lead to an increment of consumer diversity (Siemann et al. 1998; Wright and Samways 1998) because more plant species may mean more types of food, nesting sites, refuges, etc. For the present study plant richness is considered as a surrogate of habitat biotic heterogeneity (i.e. diversity of food, nesting sites, etc.).

The number of tree, shrub and herb species found within the limits (i.e. 10 × 10 m) of each of the 50 plots was counted. Plant species determinations were made by C. Ezcurra, Departmento Botánica, Universidad Nacional del Comahue.

### c) Climatic variables Temperature

Every physiological function is sensitive to temperature to some degree; this means that foraging activity tends to occur within a range of temperature for a given taxon. For example, for an “average” ant species the foraging activity peaks at around 32 °C and ceases at 40 °C (Holldobler and Wilson 1990). Among the different measures of temperatures that can be estimated (e.g. mean temperature, temperature variability) *maximum temperature* at ground level was selected to be included in the models. Maximum daily temperature shows the widest variation along the study area (14.4 – 51.6 °C) and the average temperature is different in the three habitats (Forest = 20.5 °C, Scrub = 34.8 °C and Steppe 41.8 °C). Other measures of temperature were much less variable for the study area so it was expected that maximum temperature could be more important in determining the abundance of ants.

A HOBBO H8 logger (Onset Computer Corporation, www.onsetcomp.com) was mounted on a stick a few centimeters above ground level, at the centre of each 10 × 10 m sampling plot to record the temperature every two hours during the sampling period November 2004 – March 2005. A total of 745 readings spread over a total of 62 days were obtained from the loggers that successfully remained functional until the end of the sampling season. The maximum temperatures recorded each day were recorded from these readings, and used to estimate the averaged *maximum daily temperature* (“temperature” hereafter).

### 2) Precipitation

We estimated a value of *mean annual precipitation* (“precipitation“ hereafter) for each plot by interpolation from an isoline regional map (Barros et al. 1983).

### d) Habitat use by cattle

Habitat use by large herbivores introduced to in northern Patagonia by late eighteenth century (mainly deer, cattle and horses) affect the physiognomy, composition, and several ecological processes in different habitats types (Veblen et al. 1992; Relva and Veblen 1998; Vázquez 2002). This effect cannot be neglected as a potentially important factor that could influence ant abundance-energy relationships. Also, the presence of cattle may have a positive direct effect on ant abundance, because fecal pats can provide nesting sites for *L. valdiviensis* (P. Fergnani, unpublished observations).

The amount of faeces was used as an indication of the intensity of habitat use by cattle. The number of fecal pats from cows and horses found within the limits (i.e. 10 × 10 m) of each of the 50 plots were counted as a crude measure of the level of disturbance due to habitat use by cattle. Relva and Veblen (1998) showed that, in *Austrocedrus* sp. forests in northern Patagonia, the abundance of feces can be used as an index of habitat use by herbivores as it shows a strong correlation with browsing (r = 0.65).

### Data analysis

Observed and expected frequency distribution among habitats was compared with χ^2^ test and the variation of ant abundance between habitats was tested with one-way ANOVA. *A posteriori* comparisons were performed with Fisher LSD Test.

To characterize those sites where *L. picinus* and *L. valdiviensis* were present or absent, a discriminant analysis was performed for the full data set (n = 50) for each species. Discriminant analysis was employed to identify, from a previously selected set of variables (see above), those able to characterize the distribution (i.e. presence — absence) of both species. Because the existence of colinearity among selected predictors the number of variables was pruned. First a one-way ANOVA was used to identify variables whose values differ significantly between groups (i.e. discriminate between occupied vs. unoccupied sites). Then, the inter-predictor correlations were examined to find those variables that were highly correlated (e.g. r^2^ > 0.6). Finally, redundant variables were removed, taking into account the F values (i.e. from correlated variables the one with higher F was chosen).

**Figure 3.**
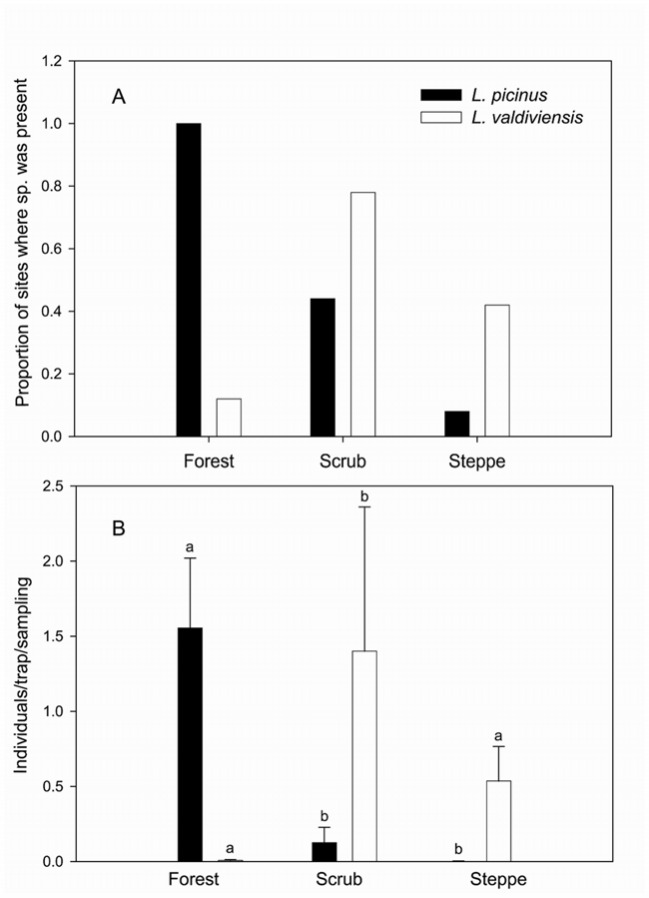
A) Frequencies and B) mean abundance of terrestrial *Lasiophanes* spp. across three habitats. Bars in B shows the standard deviation of the mean. Different letters above the bars show significant differences in mean abundance (Fisher Test p < 0.05). Comparisons should be made for each species separately.

Isoline maps were elaborated to model the spatial variation of *L. picinus* and *L. valdiviensis* abundance in the study area. Maps were also constructed for all the environmental variables included in the models. Known values of abundance (number of individuals/trap/sampling), or environmental variables at the 50 plots, were used to elaborate a continuous surface of interpolated data over the total extent of the study area using Surfer 8.0. Contour lines were obtained by the application of a geostatistical technique that uses an estimation of the semivariance of the data to perform interpolations (‘kriging’: see Matheron 1963).

Two path diagrams were elaborated to summarize in a causal scheme the relationships among environmental variables and the species abundances. Model 1 included variables linked to productivity hypothesis, and Model 2 included variables linked to resource heterogeneity. Both models also included climatic variables (precipitation and maximum daily temperature) and number of fecal pats. The relative importance of the amount of resources vs. variety of resources to explain species abundance variation was determined by comparing the two models. The first model (Model 1, Figure 5A and Figure 6A) includes tree, shrub and herb cover and ant abundance (either *L. picinus* or *L. valdiviensis*) as endogenous variables, while the second one (Model 2, Figure 5B) includes tree, shrub and herb richness and ant abundance as endogenous variables. In both models those endogenous variables depend on two climatic variables (precipitation and temperature) and the number of fecal pats. These three variables are exogenous variables in the models. A direct causal effect of precipitation and temperature is proposed on tree, shrub and herb cover (Model 1) based on evidence that suggests that changes in temperature and water availability are important ecological drivers of the spatial and temporal variation in primary productivity along the west-east gradient (Jobbágy et al. 1995; Paruelo et al. 1998a; Paruelo et al. 1998b). In the same way, changes in temperature and water availability are expected to directly affect tree, shrub and herb richness (Model 2, Hawkins et al. 2003). Hypotheses linking productivity and resource diversity to abundance (see above) justify the inclusion in this model of direct causal connections of variables representing the amount of resources available (tree, shrub and herb cover in Model 1) and variables representing variety of resources (tree, shrub and herb richness in Model 2) to ant abundance (Figure 5A & B, Figure 6). However, the direct effect of precipitation on abundance is also evaluated, as for example excessive precipitation can make foraging more difficult or low precipitation can lead to dehydration (Kaspari and Weiser 2000). The direct effect of temperature on abundance is also tested, based on the thermal limitation hypothesis. Finally, the direct and indirect causal effects of the number of fecal pats on *L. picinus* and *L. valdiviensis* abundance (through tree, shrub and herb cover in Model 1 or tree, shrub and herb richness in Model 2), are also included. It is assumed that the exogenous variables (temperature, precipitation and the number of fecal pats) may be correlated throughout.

Path analysis (PROC CALIS statement in SAS 9.1. for Windows) was used to test for the causal relationships between the abundance of *L. picinus* and *L. valdiviensis* and the environmental variables as proposed by the models. These analyses used the maximum likelihood method of parameter estimation (e.g. Hatcher 1994). The model was tested only for the occupied sites by each species (N = 23 for *L. picinus* and N = 19 for *L. valdiviensis*). All variables were standardized for the analyses. The following goodness of fit indices were used to evaluate the adequacy of the path models: 1) CFI = Bentler's comparative fit index, and 2) χ^2^, p and χ^2^/df. The CFI index provides an accurate assessment of fit regardless of sample size, and tends to be more precise than other indices (e.g. NNFI = Bender and Bonnet's non-normed fit index) in describing comparative model fit. Values of CFI over 0.9 and of χ^2^/df less than 2 are considered acceptable (see Hatcher, 1994 for references and discussion). To compare Model 1 and Model 2 for both species the overall fit was evaluated using the above described indices and the R^2^ obtained for either *L. picinus* or *L. valdiviensis* abundance.

The spatial effects were intended to be included into the analysis of abundance-environment relationships applying the method based on principal coordinates of neighbor matrices (PCNM, Borcard and Legendre 2002). Spatial filters were derived to be included in a multiple-partial regression analysis, which allows testing the partial effects of environmental variables on ant abundance by explicitly taking into account fine and broad scale spatial effects (Rangel et al. 2006). However, the spatial structure of the species abundance was not very strong and none of the derived eigenvectors were significant (e.g. r^2^ and *p* of vectors were between 0.08 and 0.005 and 0.18 and 0.75 respectively for *L. picinus* abundance and for *L. valdiviensis* abundance 0.03–0.02 and 0.43–0.52 respectively. Therefore, the partial regressions analyses were not carried out.

## Results

Thirty two ant species were captured in the pitfall traps in total, which were distributed in 10 genera and 109,000 individuals. Both *L. picinus* and *L. valdiviensis* were common species, having intermediate abundances, like other species of the genera *Camponotus, Pheidole, Solenopsis* and *Brachymyrmex*. The most abundant species were *Dorymyrmex* spp and *Acromyrmex lobicornis* while *Myrmelachista* spp showed the lowest abundances.

A total of 870 individuals of *L. picinus* and 1135 individuals of *L. valdiviensis* were caught at 46% and 38% of the 50 surveyed plots respectively. In only 7 of the 50 plots were the species found together. The distribution and abundance of species was not homogeneous across habitats. *L. picinus* was more frequently captured in the forest than in the scrub and steppe, while *L. valdiviensis* was more frequently captured in the scrub than in the other habitats (χ^2^ = 18.2, df = 2, P < 0.001 and χ^2^ = 6.91, df = 2, P = 0.03, respectively, Figure 3A). Similarly the abundance of *L. picinus* was higher in the forest than in the other habitats and the abundance of *L. valdiviensis* was higher in the scrub (F _(2, 47)_ = 18.50, P < 0.001 and F _(2, 47)_ = 4.36, P = 0.018 respectively, see Figure 3B for *posteriori* comparisons).

**Figure 4.**
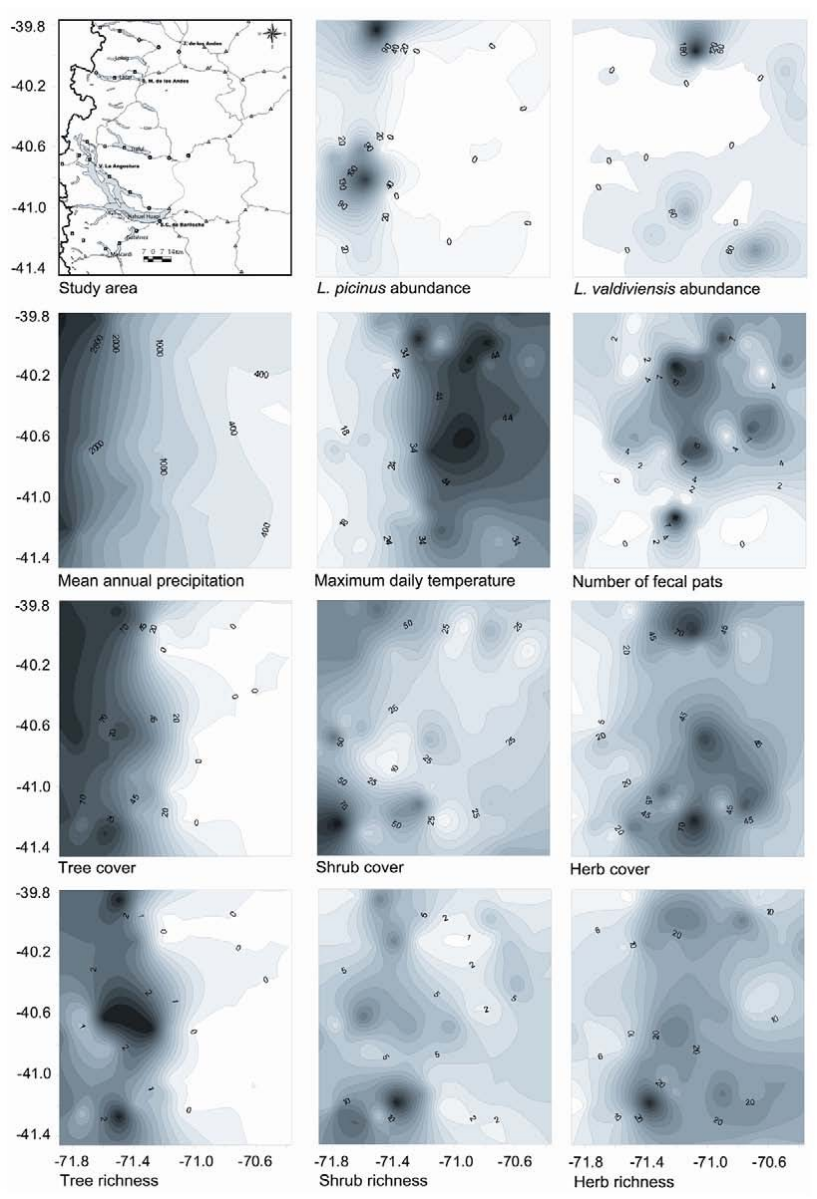
The mapping of the spatial variation of variables under study. See methods for details.

### Environmental determinants of the species distribution

The analysis reduced the list of variables to two predictors for *L. picinus* distribution: maximum daily temperature and precipitation (Standardized canonical coefficient: 0.78 for temperature and -0.36 for precipitation; Wilks' λ = 0.27, F _(2, 47)_, P < 0.000). Larger positive scores were associated with unoccupied sites where temperature is high and precipitation is low (e.g. steppes; see below) and smaller and negative scores were associated with occupied sites with low temperature and high precipitation (e.g. forests; see below). The model correctly classified 92.6 % of unoccupied sites and 91.3 % of occupied sites. For *L. valdiviensis* only herb richness was selected to be included in the discriminant analysis. The model showed 68% accuracy to discriminate between the presence and absence of both species at a site (Wilks' Lambda = 0.84, F _(1, 48)_, P < 0.005). The analysis indicated that the species were present at those sites where herb richness was higher (e.g. 18.1 species on average; see below) and absent where it was lower (12.2 species on average). The occupied sites were particularly misclassified (47.4%); while the accuracy for absences was higher (80.6%).

**Figure 5.**
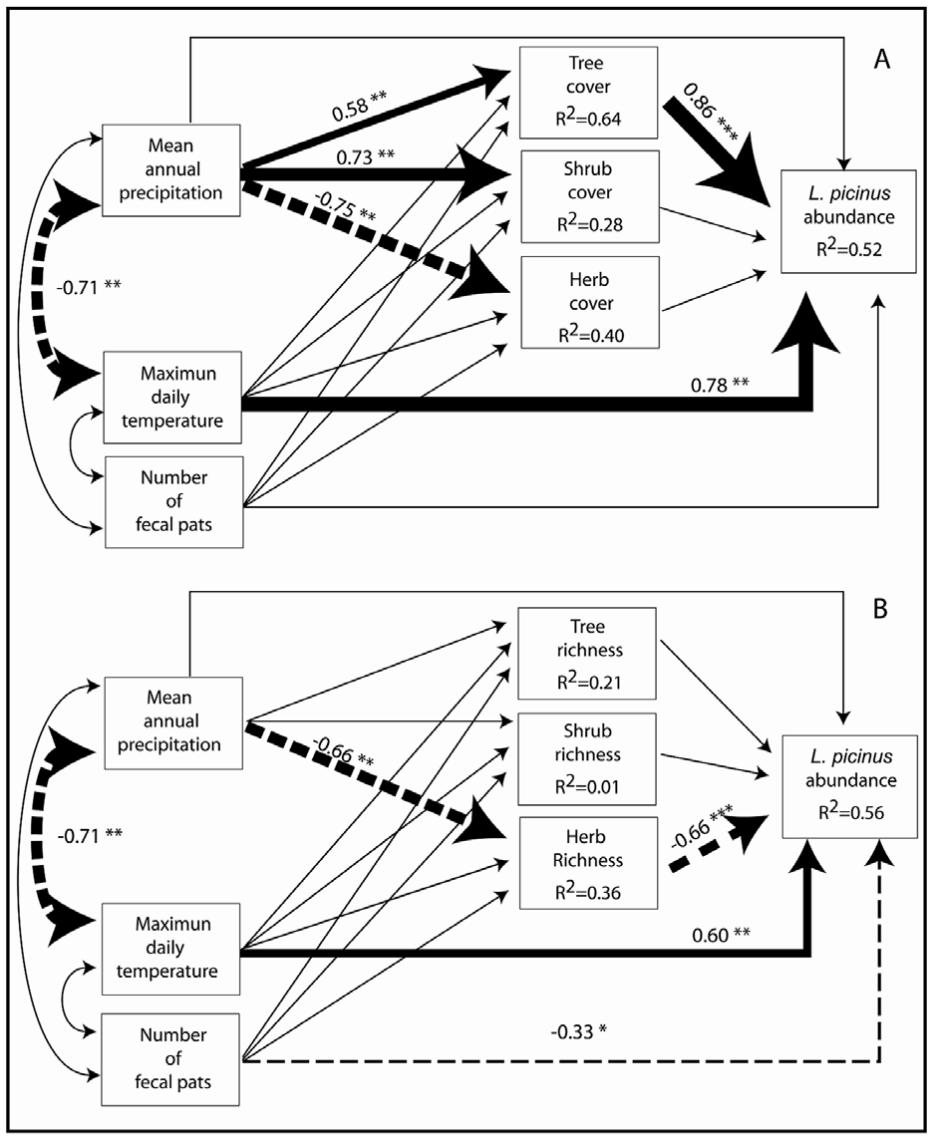
Path analysis of causal relationships among variables hypothesized to explain the variation in *L. picinus* abundance along the occupied sites. Continuous single-headed arrows or dash single-headed arrows indicate positive or negative direct effects respectively. Two-headed arrows represent correlations between exogenous variables. Only significant path or correlations coefficients are shown. The arrow line thickness is proportional to the magnitude of each significant effect to ease interpretation. The significance of the path coefficients is: *P < 0.05, ** P < 0.01, *** P < 0.001. R^2^ = coefficient of determination, indicating the proportion of variation of each endogenous variable explained by the model. A) Model 1 including plant covers, B) Model 2 including plant richness.

### Abundance variation along the environmental gradient

The mapping of the spatial variation of the species abundance shows some evidence of spatial structuring, although it was not detected by PCNM analysis; see Methods for details (Figure 4). This spatial variation involves smoothly continuous gradient-like patterns combined with some degree of geographic patchiness along the portion of the biogeographic transition zone studied. The abundance of *L. picinus* was patchy within the forests, and a rapid loss in the number of individuals caught occurred towards the east of the gradient. In contrast, the abundance of *L. valdiviensis* was patchy within the scrubs and the steppes and the loss of of the number of individuals caught occurred towards the west of the gradient. The environmental variables showed a stronger spatial structure which, in some cases, resembled that of *L. picinus* abundance, (for example, precipitation, tree cover and tree richness showed a continuous gradient-like pattern but with varying degree of patchiness within the forest), or *L. valdiviensis* abundance (for example, temperature increased toward the eastern portion of the gradient and show geographic patchiness in the scrubs and steppes region), (Figure 4). The remaining environmental variables had more idiosyncratic patterns, both with peaks in the forests (shrub cover and shrub richness) or in the scrubs and steppes (number of fecal pats, herb cover and herb richness), (Figure 4).

**Table 1.**
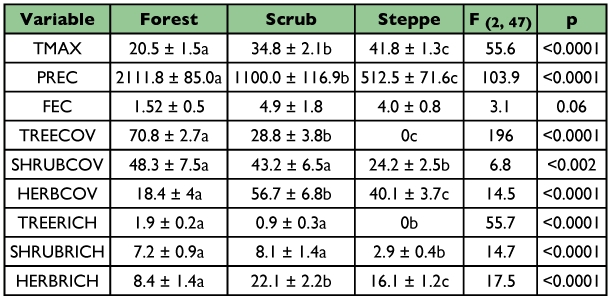
Mean values (± 1 SE) of environmental variables within three habitats. Results of one-way ANOVAs are Fisher LSD Test was used for posteriori comparisons; different letters in different habitats within a row indicates significant differences.

The averages of the environmental variables across the three habitats are shown in Table 1. The forest showed the highest values of precipitation and tree cover and the lowest values of temperature, herb cover and herb richness. The scrubs showed the highest values of herb cover and herb richness and intermediate averages for the other variables. Finally the steppes showed the highest values of temperature, and the lowest values of precipitation and tree cover.

### Environmental determinants of the species abundance

For *L. picinus* the path models suggest the presence of significant ecological structure in the abundance-environment relationship (Figure 5A, B). Model 1 (which includes variables linked to productivity hypothesis) showed a good fit to the data (χ^2^ = 1.57, df = 3, P = 0.66, χ^2^/df = 0.52, CFI = 1) and explained 52% of mean variation in abundance (Figure 5A). Model 2 (which includes variables linked to resource heterogeneity) showed a reduced fit to the data (χ^2^ = 6.18, df = 3, P = 0.10, χ^2^/df = 2.1, CFI = 0.91) and explains 56% of the species abundance variation (Figure 5B). The increase of *L. picinus* abundance in Model 1 was associated with both to a direct and indirect effect of climate and the presence of forests towards the west of the gradient. Only maximum daily temperature, an exogenous variable, showed a direct effect on *L. picinus* abundance with increasing abundance with higher temperature. Precipitation had an indirect effect on *L. picinus* abundance mediated mainly by tree cover. The number of fecal pats showed no effect. Of the endogenous variables only tree cover showed a significant effect on *L. picinus* abundance, increasing abundance with higher canopy cover (Figure 5A).

**Figure 6.**
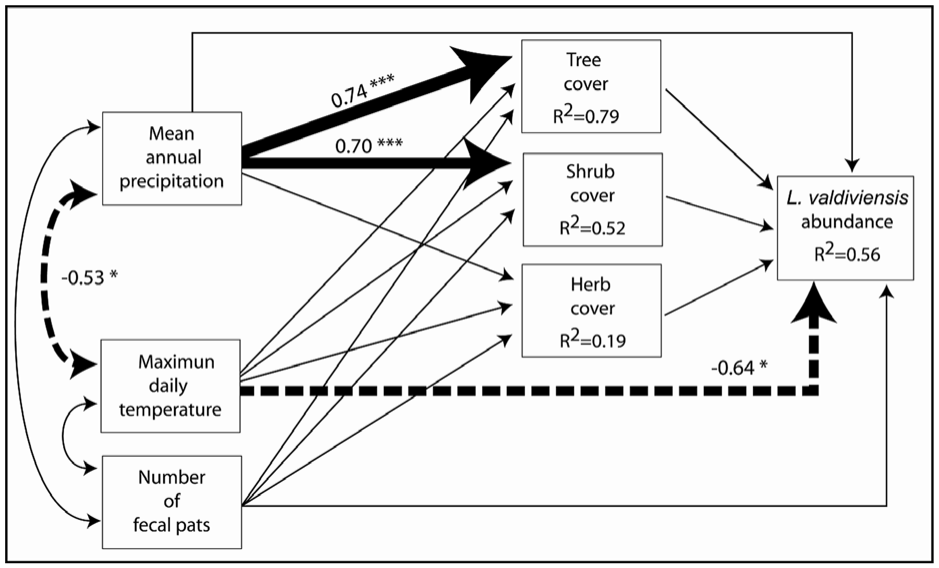
Path analysis of causal relationships among variables hypothesized to explain the variation in L *valdiviensis* abundance along the occupied sites (Model 1 including plant covers). Continuous single-headed arrows or dash single-headed arrows indicate positive or negative direct effects respectively. Two-headed arrows represent correlations between exogenous variables. Only significant path or correlations coefficients are shown. The arrow line thickness is proportional to the magnitude of each significant effect to ease interpretation. The significance of the path coefficients is: *P < 0.05, ** P < 0.01, *** P < 0.001. R^2^ = coefficient of determination, indicating the proportion of variation of each endogenous variable explained by the model.

Model 2 has to be interpreted with caution given that it showed a marginal fit to data. When plant richness is considered, the increase of *L. picinus* abundance was associated to direct and indirect effect of climate, direct effect of cattle and the effect of herbaceous plants variety. Temperature has a direct and positive effect on *L. picinus* abundance, while precipitation has a positive indirect effect on *L. picinus* abundance mediated by herb cover. *L. picinus* abundance decreases with both the increment of the number of fecal pats and herb richness (Figure 5B).

For *L. valdiviensis* only Model 1 showed a good fit to the data (χ^2^ = 4.67, df = 3, P = 0.20, χ^2^/df = 0.52, CFI = 0.96) and explains 36% of mean variation in abundance of both species (Figure 6). Model 2 showed a poor fit and was rejected (χ^2^ = 11.00, df = 3, P = 0.20, χ^2^/df = 3.67, CFI = 0.69). In Model 1, climate showed a strong direct and negative effect on *L. valdiviensis* abundance, while no effect of plant cover was detected. *L. valdiviensis* abundance was higher where temperature was low. Although precipitation positively affects tree and shrub cover, none of these productivity- related variables significantly affects *L. valdiviensis* abundance.

## Discussion

### The species distribution

The distribution of *L. picinus* and *L. valdiviensis* along the environmental gradient is different. *L. picinus* inhabits principally the forests whereas *L. valdiviensis* makes major use of the scrubs; this is in the transition area between forests and steppes. Probably, the sharp W-E environmental gradient has played a role in the appearance and/or maintenance of different *Lasiophanes* species in this region. It has been proposed that adaptive variation is usually well represented along environmental transitions, and that environmental gradients are important in diversification and speciation (see Ruggiero and Ezcurra 2003 for general discussion and examples).

The combination of two environmental factors, maximum temperature and precipitation, predicted the distribution of *L. picinus* along the environmental gradient with a certainty higher than 90%. These two variables are good descriptors of macrohabitat (forest, scrub, and steppe) along the studied area (see Table 1). Because this species is mainly confined to the forests, which is a habitat that develops where the average temperature is lowest and average precipitation is highest, the distribution of *L. picinus* can be well predicted by those factors only. On the other hand, the distribution of *L. valdiviensis*, a species principally captured in the scrubs, could be predicted with only 70% accuracy. In comparison to the forests and steppes, which can be accurately described, for example, forests have high tree cover and steppes have a high proportion of bare soil (Table 1), the scrubs possess intermediate values for the majority of the environmental variables analyzed. Only herb cover and herb richness had the highest values in the scrubs. Therefore, the inclusion of herb richness in the discriminant model allows prediction of the distribution of *L. valdiviensis*. It is probable that the combination of reduced shade in comparison to the forest, and the higher precipitation compared to the steppes, increase the abundance and richness of herbs in the scrubs.

### The variation of the species abundance

Among occupied sites, Model 1, which relates to the amount of resources, explained a greater proportion of the variation of the abundance for *L. picinus* than for *L. valdiviensis* (52% and 36% respectively). The fact that *L. picinus* is distributed in places of more homogeneous characteristics along the environmental gradient than *L. valdiviensis* may have affected the results. *L. picinus* was captured at all forest sites and in some “forest-like” scrub sites where tree cover was around 50%. On the other side, *L. valdiviensis* was more frequently captured in the scrubs but was also present in the steppes as well. According to the environmental variables considered in this study the scrubs and forest are more alike than the scrubs and steppes (see Table 1). This might make it more difficult to explain the abundance variation of a species that inhabits scrubs and the steppes than that of a species that inhabits the forest and scrubs. Also, within the study area the environmental variables analyzed showed more idiosyncratic patterns within the scrubland-steppes than within the forests (Speziale 2006) (Figure 4).

Nevertheless, the proportion of variance explained for both species is quite low, indicating that other variables not included in our analyses may contribute to abundance variation (for example, biotic interactions and soil attributes). For example, Kusnezov (1952) pointed out the presence of homopterans inside the nests of *L. picinus* that are associated with plant roots. The author mentioned that there is probably a relationship between ants and homopterans as it also occurs with *Lasius* in the Northern hemisphere. Also, it is well known that for ant assemblages there is an important relationship between environmental conditions such as habitat favorability, and dominance with species richness and abundance (Andersen 1992). Indeed, in NW Patagonia, habitat condition has a great influence on the dominant species abundance (e.g. *Dorymyrmex* spp) which in turn affects subordinated species abundance and richness (Farji-Brener et al. 2002; Sackmann and Farji-Brener 2006).

Although we have analyzed the climatic variables usually selected to predict ant abundance at different scales (i.e. productivity and temperature), seasonality (the number of months with mean temperature less than 0 °C) was not considered. Kaspari et al. (2000a) showed that for a given mean temperature, nest density at geographic scale was higher in seasonal sites with longer and colder winters than in sites with a more even temperature regime. They argued that seasonality could benefit ectotherms that use winters as metabolic refugia. This may be the case for *Lasiophanes* species, which are social insects that nest in sheltered sites and that overwinter. The relationship between this climatic variable and the ecology of these species may also be important in regulating their population size.

Among the variables included in the path models, the maximum temperature was shown to be a key variable in explaining the variation in abundance of both species. The effect of temperature on abundance of both species was direct but the sign of the relationship was different. The increment of temperature implies an increase of *L. picinus* abundance but a decrease of *L. valdiviensis* abundance. The reason for this result may be that the species use different habitats. *L. picinus* inhabits predominantly humid forests where temperature is indeed reduced, thus the increment of maximum daily temperature may lead to an increment of worker activity that increases pitfall captures. On the other hand, Kusnezov (1959) mentioned that *L. valdiviensis* can inhabit arid areas but only occupies sites where the effects of droughts are ameliorated by factors like the presence of cushion-like plants like *Azorella* spp under which ants can nest. This means that in some way the species is avoiding extreme xeric conditions and that temperature can have an effect that is opposite from that predicted by classical thermal limitation hypothesis (Kaspari et al. 2000a), at least at the scale of this study.

In general, our findings support energy theory and agree with previous investigations carried out for ants (Hawkins et al. 2003; Kaspari et al. 2004). For example, temperature explained significant proportions of the variation in ant species density and abundance in the Northern Cape Floristic Region (Botes et al. 2006). Also, nest density increased with mean temperature at a geographical scale (Kaspari et al. 2000a).

Mean annual precipitation, the other climatic variable examined, never showed a direct effect on ant abundance. Our results confirmed that the increase in precipitation has a strong positive influence on plant productivity within the Patagonian scrubland-steppes (see Jobbágy et al. 1995). However, only tree cover translated into an increase in *L. picinus* abundance (see below). The indirect effect of precipitation mediated by tree and shrub cover on *L. valdiviensis* is low, as the path coefficients for plant cover and *L. valdiviensis* abundance were not significant. We expected that variation in precipitation, which is considerable across the studied biogeographical transition, would have a substantial influence on variation in species abundance. For example, in water limited systems such as the steppes in the present study, water availability is much more likely to be a strong correlate of richness than is absolute energy availability (Hawkins et al. 2003). It is probable that precipitation is not a limiting factor within the forest and scrub habitats occupied by the species, although it is highly correlated to plant cover.

Tree cover was found to be strongly associated with *L. picinus* abundance. While this species does not feed or nest in trees, it may make use of fallen branches and decaying wood to nest, variables which are certainly correlated to tree cover. Also, tree cover determines specific microclimatic conditions such as increased shade, amount of litter, soil humidity, etc., that in turn may affect the species activity.

Overall, these results showed that the amount of resources (i.e. Model 1) seemed to be more important than the variety of resources to explain the variation of the species abundance since Model 1 had better fit to data than Model 2. Furthermore, for *L. picinus*, the number of fecal pats appeared to have an effect in Model 2 only when the variables related to plant cover are replaced by variables with lower explanatory power, such as plant richness. These results are in keeping with the prediction derived from the productivity hypothesis that more resources mean increased abundance. However, it is also probable that plant cover, not only provides the species with more resources but can also determine the habitats microclimatic conditions as discussed above. A deeper knowledge of the ecology of the species, would allow having a clearer interpretation for this result.

Herb richness was found to negatively affect *L. picinus* abundance (Model 2). A satisfying explanation for this direct effect was not apparent. We suspect that this relationship reflects habitat preferences of the species rather than a true relationship between plant richness and *L. picinus* abundance. On the one hand, herb richness was important where precipitation and tree cover are low (Figure 4). On the other hand, tree cover had a direct positive effect on *L. picinus* abundance. Thus it is possible that the link between herb richness and abundance is mainly related to site characteristics such as high number of herb species and low canopy cover.

Changes in ant abundance due to the presence of cattle are often attributed to the modifications that grazing causes to vegetation. For example, the whole vegetation strata can be removed by grazing, leaving habitats with little or no understory or ground vegetation. Also, grazing can prevent regeneration of woody tree species and other native vegetation, and may increase the vulnerability of the area to weed invasion (Hobbs 1987; Saunders et al. 1991). However, no effects were found of the number of fecal pats on plant cover or plant richness, so it is likely that the presence of cattle themselves affected the abundance of *L. picinus*. But, the presence of cattle may not only affect vegetation, they may also deteriorate the soil structure as well. Cattle activity can decrease the infiltration capacity of the soil, increase runoff, and promote soil erosion and thus modify the ground microclimate, change soil moisture content, level of insolation, humidity and exposure (King and Hutchinson 1983; Abbot 1989; Neave and Tanton 1989). *L. picinus* nests mainly in the soil and thus the presence of cattle by altering soil attributes might change the nesting success of the species. The nature of the relationship of the number of fecal pats with *L. picinus* abundance cannot be assessed because variables associated to soil attributes have not been measured. It may also happen that cattle trampling and wallowing cause the destruction of nests and consequently a decrease in the number of workers in the pitfall catches.

The present work provides new ecological data on a poorly known endemic ant genus of Patagonia. In particular, for *L. valdiviensis* (*sensu stricto)* this is the only work available on its ecology and environmental requirements. In addition, we provide evidence that changes in temperature (e.g. global climate change) may have important consequences on these species populations as it has been suggested for other insect species (Hodkinson et al. 1999; Kaspari et al. 2000a; Botes et al. 2006).
